# Organophosphorous Pesticide Detection in Olive Oil by Using a Miniaturized, Easy-to-Use, and Cost-Effective Biosensor Combined with QuEChERS for Sample Clean-Up

**DOI:** 10.3390/s17010034

**Published:** 2016-12-24

**Authors:** Fabiana Arduini, Matteo Forchielli, Viviana Scognamiglio, Kozitsina Alisa Nikolaevna, Danila Moscone

**Affiliations:** 1 Department of Chemical Science and Technologies, University of Rome “Tor Vergata”, Via della Ricerca Scientifica, 00133 Rome, Italy; matteo.forchielli@gmail.com (M.F.); moscone@uniroma2.it (D.M.); 2 Institute of Crystallography (IC-CNR), Via Salaria km 29.300, 00015 Monterotondo, Italy; viviana.scognamiglio@mlib.ic.cnr.it; 3 Department of Analytical Chemistry, Institute of Chemical Engineering, Ural Federal University Named After the First President of Russia B. N. Yeltsin, Mira 19, 620002 Yekaterinburg, Russian Federation; a.n.kozitsina@urfu.ru

**Keywords:** organophosphorous pesticides, electrochemical biosensor, butyrylcholinesterase, carbon black, screen-printed electrodes, QuEChERS

## Abstract

Herein, we report a portable electrochemical biosensor based on butyrylcholinesterase (BChE) immobilized on carbon black (CB)-modified screen-printed electrodes (SPEs) for the detection of organophosphorous pesticides in olive oil. The BChE/CB-SPE biosensor was developed to detect paraoxon in standard solutions as well as in olive oil samples previously treated with the QuEChERS method to extract pesticides from the whole fatty matrix. The biosensor shows a linear concentration range of between 20 and 100 ppb for paraoxon both in standard solutions (phosphate buffer 0.05 M) and in olive oil extracts, with a detection limit of 6 ppb in olive oil extract, corresponding to 10% of inhibition. The accuracy of this biosensor in olive oil samples was assessed with olive oil spiked with paraoxon, obtaining satisfactory recovery values.

## 1. Introduction

Olive oil represents an important agricultural product in terms of well-being and economy in Europe as well as around the world for its nutritional, health, and sensory properties [1]. The European Union (EU) is the leading world producer, accounting for 80% and consuming 70% of olive oil. In this context, the main aim of EU policy is to maintain and strengthen its position in world markets by encouraging production of a high-quality product, simplifying the rules, and implementing more effective monitoring for both quality and safety control. The Commission Regulation (EC) No. 1183/2008 of 28 November 2008 amending Regulation (EC) No. 1019/2002 on marketing standards for olive oil [2] lays down the standards for labeling, advertising, packaging, and presentation required for marketing in the EU. This regulation provides important safeguards for the consumer and allows the producers to maximize the benefits of selling on the basis of quality. Furthermore, the Codex Alimentarius Commission established several regulations to set maximum residue limits (MRLs) in food, including olives and olive oil [3].

Organophosphates are one of the major classes of pesticides employed in the agrifood sector due to their high toxicity but relatively low persistence in the environment. This can cause the presence of trace amounts of these compounds in olive oil samples; thus, their absence at the legal limit constitutes an important parameter of the product’s quality. Because the toxic effect that these substances may exert on human well-being, MRLs at mg/kg for pesticide residues in several foods including virgin olive oil have been established [4]; for instance, for organophosphate fenthion the MRL was set at 1 mg/kg [3]. Due to the severe problem of pesticide contamination, the European Commission has planned a coordinated multiannual control program for 2015, 2016, and 2017 to ensure compliance with maximum residue levels of pesticides and to assess consumer exposure to pesticide residues in and on foods of plant and animal origins. In this regulation, virgin olive oil is presented as a food, while several organophosphates (e.g., azinphos-methyl, chlorpyriphos, diazinon, dichlorvos, ethion, fenthion, malathion, parathion, pirimiphos-methyl) are selected as pesticides [5]. Besides food contamination, pesticides are also considered as priority hazardous substances in the field of water policy, owing to their prevalent use in agrifood [6]. Indeed, entering into the food chain, they are able to contaminate air, water, and soil, affecting ecosystems and producing health complications in animals and humans.

Conventional analytical methods used for pesticide detection (e.g., paraoxon) are based on gas chromatography; however, these methods require a laboratory set-up, expensive instrumentation, skilled personnel, and long procedures for sample treatment. In this context, the availability of analytical devices such as bioassays and biosensors able to establish the high quality and safety of olive oil products has become a crucial requirement, especially in foodstuffs with a high fat content where interfering species often mask the signal of the target compounds. Without a doubt, the analysis of pesticides in olive oil is very challenging because of the inherent complexity of the matrix, mainly consisting of triglycerides (98%–99%). This drives the need for different strategies to isolate or extract the pesticide fraction from the whole fatty matrix, as pesticides are non-polar, fat-soluble compounds. These strategies include laborious and time-consuming clean-up techniques, necessary in the case of subsequent analytical processes, to separate the low-molecular-mass pesticides from the higher-molecular-mass fat, including liquid-liquid partition, gel permeation chromatography, solid-phase extraction, and matrix solid-phase dispersion. 

A widely employed methodology for sample treatment was described by Anastassiades et al. [7]. This method, called QuEChERS (Quick, Easy, Cheap, Rugged and Safe), is based on liquid-liquid partition with acetonitrile followed by a clean-up step with dispersive SPE, where a mixture of different sorbents (C18, primary secondary amine (PSA), and graphitized carbon black (GCB)) is used instead of PSA alone. The main advantages of this method are its simplicity, the use of cheap disposable reagents and materials, the small volume of organic solvent consumed, and the large number of samples that can be processed in an hour.

Herein, we demonstrated the suitability of the well-known QuEChERS method as a sample treatment for complex food matrices in combination with a miniaturized, easy-to-use, and cost-effective electrochemical biosensor for paraoxon detection in olive oil, based on the enzyme butyrylcholinesterase (BChE) immobilized on carbon black nanomaterial (CB)-modified screen-printed electrodes (SPEs). The use of BChE as a bioreceptor for pesticide electrochemical monitoring has been widely documented in previous articles, where its high robustness and reliability were extensively demonstrated [8,9]. CB is likewise recognized as a cost-effective nanomaterial (only 1 euro for 1 kg of CB) to modify screen-printed electrodes, as it is able to enhance the electrochemical properties of the electrodes [10,11,12,13,14,15,16].

Actually, very few articles have been reported in the literature describing biosensors/bioassays for pesticide detection in olive oil; however, multistep procedures, including sample treatment with heating and liquid-liquid extraction, were necessary for the analysis [17,18]. In this context, the novelty of the presented work resides in the combination of the QuEChERS method with the well-established BChE/CB-SPE biosensor for pesticide detection in such complex matrices as olive oil, demonstrating the possibility to exploit an easy sample treatment in combination with a smart biosensor for fast and cost-effective pesticide extraction/detection in olive oil.

## 2. Materials and Methods

### 2.1. Reagents and Apparatus

Commercial CB N220 of industrial standard grade was obtained from Cabot Corporation (Ravenna, Italy). Butyrylcholinesterase (BChE) from equine serum, bovine serum albumin (BSA), S-butyrylthiocholine chloride, 5,5’-dithio-bis-(2-nitrobenzoic acid) (DTNB), glutaraldehyde and paraoxon (paraoxon-ethyl), Nafion^®^ (perfluorinated ion-exchange resin, 5% *v*/*v* solution in lower alcohols/water), acetonitrile, formic acid, ammonium formate (all HPLC grade) were purchased from Sigma Aldrich Company (St. Louis, MO, USA). Amperometric measurements were carried out using a portable PalmSens (Palm Instruments^®^, Houten, The Netherlands).

### 2.2. Preparation of SPE

SPE was produced with a 245 DEK (Weymouth, MA, USA) screen-printing machine according to the procedure previously reported [11]. 

### 2.3. Preparation of BChE Biosensor

The biosensor was prepared immobilizing BChE on SPE modified with CB according to the procedure previously reported [8,9].

### 2.4. Paraoxon Determination Using Biosensor

The inhibitory effect of paraoxon on BChE biosensor was evaluated by determining the decrease in the current obtained for the oxidation of the thiocholine produced by the enzyme. A drop (50 μL) of buffer solution (0.05 M + 0.1 M KCl, pH 7.4) containing different amounts of butyrylthiocholine was placed onto the BChE biosensor covering working, counter, and reference electrodes. After applying the potential (+300 mV vs. Ag/AgCl), the signal was continuously recorded and the current value at the steady state was detected (after 5 min). Then, BChE biosensor was incubated in 50 μL of paraoxon solution for 20 min and then rinsed with distilled water. After that, the response toward the substrate was again registered and the degree of inhibition was calculated as a relative decay of the biosensor response Equation (1).

I% = [(I_0_ − I_i_)/I_0_] × 100 (1)

where I_0_ and I_i_ represent the biosensor response before and after the incubation procedure, respectively. Paraoxon inhibition as well as acetonitrile effect was evaluated with the same procedure. To evaluate the amount of paraoxon in olive oil, after the treatment with QuEChERS the sample was dried using the instrument Reacti-ThermTM III Heating Modules, and thus solubilized in buffer 0.05 M + KCl 0.1 M pH = 7.4 + acetonitrile 10% (*v*/*v*).

### 2.5. Paraoxon Measurement in Olive Oil Extract Using HPLC/MS

High Performance Liquid Chromatographic coupled with Mass Spectrometer Analyzer (HPLC/MS) was used for paraoxon measurements in olive oil. In detail, the following instruments were employed: Agilent Technologies 1290 Infinity pump, Agilent Technologies 1290 Infinity autosampler, Agilent Technologies 1290 Infinity termostat, Agilent Technologies 6410 Triple Quad LC/MS, chromatographic column Agilent Eclipse Plus C18 RRHD 1.8 μm 2.1 × 100 mm. The olive oil extracted samples (5 µL) were injected in the column and a mobile phase consisting of two components was used: (A) Acetonitrile + 0.1% Formic acid; and (B) H_2_O + 0.1% Formic acid + 5 nM Ammonium Formate, applying the following gradient: 0–1 min 95% B and 5% A; 2–13 min 95% A and 5% B. The flow rate was 0.18 mL/min and the column was thermostated at 35 °C.

## 3. Results and Discussion

In this work, a BChE/CB-SPE biosensor was developed to detect paraoxon in standard solutions as well as in spiked olive oil samples, previously treated with the QuEChERS method to extract the pesticide fraction from the whole fatty matrix. 

The use of BChE as a stable and reliable bioreceptor has been widely described in the literature [8,9,19,20]. BChE is irreversibly inhibited by organophosphorous and carbammic pesticides; thus, measuring the enzyme activity before and after the biosensor’s exposure to contaminated samples, it is possible to quantify the amount of these pesticides in a concentration-dependent manner.

This BChE biosensor was chosen taking into account the high sensitivity and storage stability at room temperature in dry conditions [19], thanks to the ad hoc configuration of the bioreceptor immobilized on a tailored electrochemical surface. Moreover, the use of CB allowed us to easily prepare a stable and cost-effective dispersion suitable for mass-produced modified sensors (e.g., using BioDot dispensing equipment), which is able to sensibly decrease the applied potential, making the thus-assembled biosensor competitive in comparison to the ones reported in the literature [21,22,23,24]. Paraoxon has been selected as a model compound of organophosphorus pesticides to assess the effectiveness of the proposed biosensor, since it is usually exploited for this kind of measurement [8,9,19,20].

**Figure 1:**
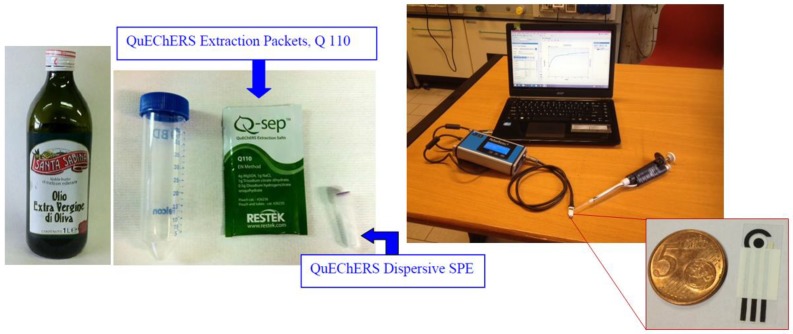
Portable electrochemical biosensor based on butyrylcholinesterase (BChE) immobilized on carbon black (CB)-functionalized screen-printed electrodes (SPEs) combined with QuEChERS extraction packets to extract pesticides from olive oil.

Moreover, we demonstrated the suitability of the QuEChERS method, widely employed for pesticide determination, using reference chromatographic techniques, as a sample treatment to extract organophosphorous pesticides from a complex food matrix such as olive oil, for its successive analysis through a miniaturized, easy-to-use, and cost-effective electrochemical BChE biosensor immobilized on CB-SPEs. The whole system is reported in Figure 1.

### 3.1. Optimization of Paraoxon Analysis in Standard Solutions

**Figure 2:**
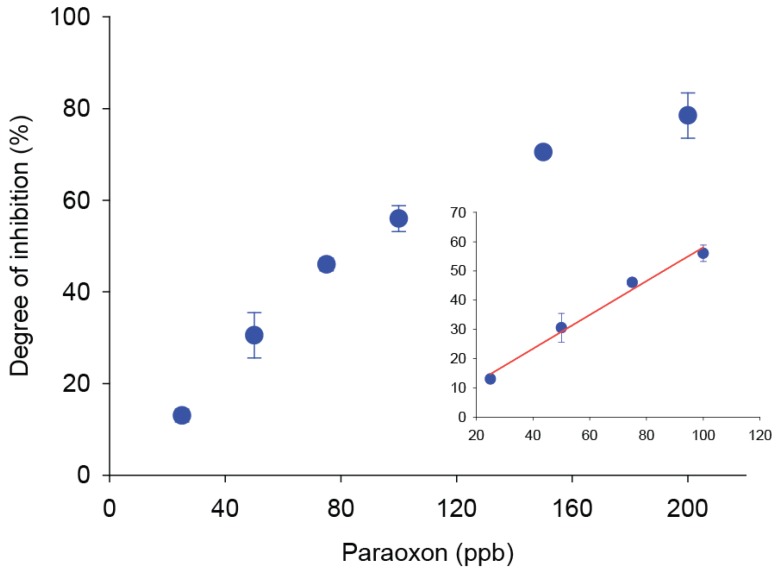
Calibration curve of paraoxon. Substrate: 5 mM butyrylthiocholine in phosphate buffer 0.05 M + KCl 0.1 M pH = 7.4, incubation time 20 min, applied potential +300 mV vs. Ag/AgCl (*n* = 3). Inset: Linear range of the calibration curve.

The BChE/CB-SPE biosensor was optimized for paraoxon detection in standard solution through a drop analysis, with the aim to tailor the measurements for the small volume obtained after QuEChERS extraction. According to our previous results obtained using a BChE/CB-SPE biosensor for pesticide detection in water samples [8], a concentration of the BChE substrate (butyrylthiocholine) of 5 mM was chosen to be a minimum amount to reach the V_max_, considering that paraoxon is capable of irreversibly inhibiting BChE. An incubation time of 20 min was also chosen as a compromise between a sensitive measurement and a reasonable time of analysis [20]. Thus, standard solutions of paraoxon in a concentration range of 20–200 ppb were analyzed, and a calibration curve is reported in Figure 2.

The inhibition percentage was calculated using the “medium exchange method” to avoid both electrochemical and enzymatic interferences. Indeed, according with this procedure, the electrochemical interferences are eluded since the residual enzymatic activity is measured in a new phosphate buffer solution in the absence of the real sample. Enzymatic interferences such as reversible inhibitors (e.g., fluoride) [25,26] and detergents [27] or heavy metals such as Pb^2+^ and Zn^2+^ [28,29] are avoided by carefully washing the biosensor with distilled water after the inhibition step. In this way, only inhibitors covalently bound to the enzyme are measured, including irreversible inhibitors such as paraoxon.

In the calibration curve described in Figure 2, a linear range was observed between 20 and 100 ppb range, described by the equation *y* = (0.62 ± 0.03) *x* − (1.9 ± 2.1) with a R^2^ = 0.986 (Figure 2, inset).

### 3.2. Inactivation Studies in the Presence of Acetonitrile

**Figure 3:**
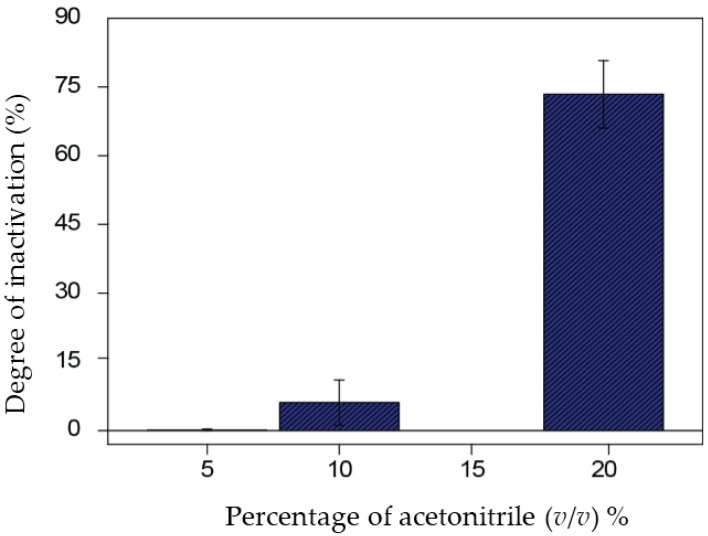
Degree of inactivation recorded for 5 mM butyrylthiocholine in phosphate buffer 0.05 M + KCl 0.1 M pH = 7.4, in the presence of different amounts of acetonitrile (*n* = 3).

Since the extraction procedure of pesticides from olive oil samples employs organic solvents (i.e., acetonitrile), it was necessary to evaluate its effect on BChE activity in order to demonstrate that the inhibition effect was due to the presence of pesticides and not to the organic solvent. Thus, the effect of acetonitrile at different concentrations was evaluated. As shown in Figure 3, acetonitrile in the range between 5% and 10% (*v*/*v*) can be used to perform pesticide extraction without interfering with paraoxon measurements. An acetonitrile concentration of 10% was selected, in agreement with the colorimetric AChE assay reported by Oujji et al. [18], demonstrating the robustness of the cholinesterase-based bioassay also using different configurations.

### 3.3. Inhibitory Effect of Paraoxon Extracted from Olive Oil Samples

In order to evaluate the applicability of this biosensor in real samples, the suitability of the system was evaluated for the detection of paraoxon in olive oil, analyzing the matrix effect and recovery.

**Figure 4:**
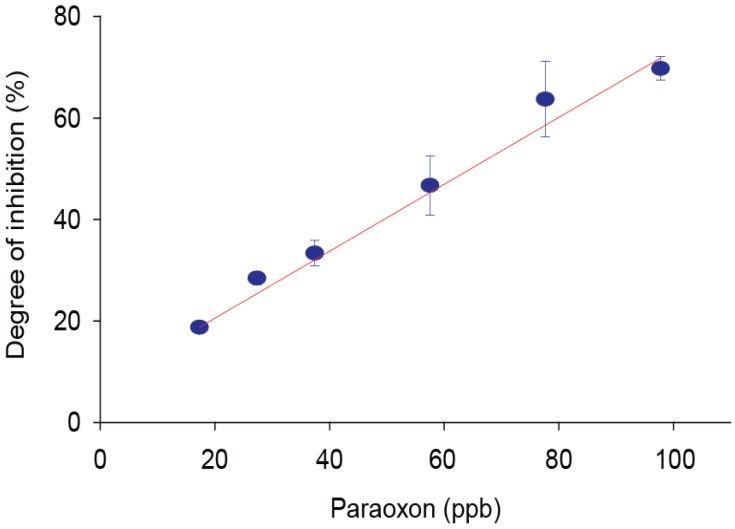
Olive oil matrix effect. Inhibition percentage of the current signal recorded in the presence of different concentrations of paraoxon in olive oil extracted with QuEChERS.

To estimate the matrix effect, a real sample of olive oil was treated with the QuEChERS method and the extract was fortified with paraoxon in a concentration range of 20–100 ppb and then measured. A calibration curve was thus constructed (Figure 4) measuring the inhibition percent. A linear range was obtained between 20 and 100 ppb range, described by the equation *y* = (0.65 ± 0.03) *x* + (5.8 ± 0.7) with a R^2^ = 0.963. A detection limit of 6 ppb was assessed, corresponding to 10% of inhibition. The slope of the calibration curve is practically equal to the one obtained from the buffer solution, demonstrating the absence of the matrix effect.

This calibration curve was further used as a reference to calculate the recovery values of the olive oil sample. The results obtained, adding paraoxon to a commercial extra virgin olive oil before the QuEChERS treatment, are reported in Table 1.

The medium recovery for paraoxon from olive oil samples (60%) was similar to the recovery evaluated with HPLC (63%). These results are in excellent agreement with recovery values usually obtained using QuEChERS extraction methods combined with a chromatographic method, as reported in the QuEChERS informational booklet for paraoxon extraction [30]. These low recovery values are ascribed to the QuEChERS extraction method which, in the case of paraoxon, are smaller in comparison with other organophosphorous pesticides, which can be extracted with higher values (e.g., dichlorvos ca. 80% and chlorpyrifos ca. 75%). In any case, this wide range of values is due to the high complexity of the olive oil matrix where recovery values should be fall in the range of 70%–120% [31]. However, we found comparable recovery values obtained using both HPLC and the proposed biosensor, demonstrating the suitability of this biosensor even in a complex matrix such as olive oil. Furthermore, the storage stability of this biosensor was tested at room temperature under dry conditions up to 60 days with 80 ppb of paraoxon, obtaining the same degree of inhibition within relative standard deviation (RSD%) in the entire tested period (data not shown). The results achieved demonstrated high storage stability in agreement with our previous results [8].

## 4. Conclusions

The development of an electrochemical biosensor based on BChE immobilized on CB-functionalized SPEs led to the determination of paraoxon in a complex matrix such as olive oil after a simple QuEChERS extraction, avoiding laborious pre-treatments. The BChE/CB-SPE biosensor presented a good reproducibility as well as good analytical performances with a low detection limit (6 ppb) in olive oil extract. The simplicity and reliability of this biosensor along with the advantages of fast response and extended storage stability, which are key points for a successful commercialization, make this analytical system very attractive for pesticide detection in olive oil. Taking into account the MRL established for pesticides in olive oil at mg/kg levels including organophosphates [3], the proposed sensor can be considered a suitable analytical tool for the analysis of these contaminants in olive oil.

Furthermore, the proposed biosensor is competitive since very few examples of cholinesterase-based tools for pesticide detection in olive oil have been described in the literature, with the exception of the biosensor/bioassay reported by Noguer’s group [17,18], focused on malathion, methidathion, and dimethoate in their oxidized forms, which requires a simple sample treatment but is not suitable for successive chromatographic analyses. Indeed, the novelty of this work resides in the combination of the QuEChERS method with the well-established BChE/CB-SPE biosensor for pesticide detection in complex matrices such as olive oil, proving the ability to use an easy sample treatment with fast and cost-effective pesticide extraction/detection. The benefit is that, as QuEChERS is the same method exploited for standard analysis, only samples that are positive for the presence of pesticides should be further analyzed with chromatographic techniques without additional clean-up procedures.

## Figures and Tables

**Table 1:** Recovery study of paraoxon taken as a model compound of organophosphorus pesticides (*n* = 3).

Sample	Paraoxon Added (ppb)	Paraoxon Detected (ppb)	Recovery % Biosensor	Recovery % HPLC
extra virgin olive oil	100	60 ± 2	60 ± 2	63 ± 2
